# Genome-Wide CRISPR Screen Reveals Cancer Cell Resistance to NK Cells Induced by NK-Derived IFN-γ

**DOI:** 10.3389/fimmu.2019.02879

**Published:** 2019-12-11

**Authors:** Xiaoxuan Zhuang, Daniel P. Veltri, Eric O. Long

**Affiliations:** ^1^Laboratory of Immunogenetics, National Institute of Allergy and Infectious Diseases, National Institutes of Health, Rockville, MD, United States; ^2^Bioinformatics and Computational Biosciences Branch, Office of Cyber Infrastructure and Computational Biology, National Institute of Allergy and Infectious Diseases, U.S. National Institutes of Health, Rockville, MD, United States

**Keywords:** natural killer cells, MHC class I, IFN-γ, IFNGR2, CRISPR screen, anti-leukemia activity

## Abstract

The anti-leukemia activity of NK cells helps prevent relapse during hematopoietic stem cell transplantation (HSCT) in leukemia patients. However, the factors that determine the sensitivity or resistance of leukemia cells in the context of NK-mediated cytotoxicity are not well-established. Here, we performed a genome-wide CRISPR screen in the human chronic-myelogenous-leukemia (CML) cell line K562 to identify genes that regulate the vulnerability of leukemia cells to killing by primary human NK cells. The distribution of guide RNAs (gRNAs) in K562 cells that survived co-incubation with NK cells showed that loss of *NCR3LG1*, which encodes the ligand of the natural cytotoxicity receptor NKp30, protected K562 cells from killing. In contrast, loss of genes that regulate the antigen-presentation and interferon-γ-signaling pathways increased the vulnerability of K562 cells. The addition of IFN-γ neutralizing antibody increased the susceptibility of K562 cells to NK-mediated killing. Upregulation of MHC class I on K562 cells after co-incubation with NK cells was dependent on *IFNGR2*. Analysis of RNA-seq data from The Cancer Genome Atlas (TCGA) showed that low *IFNGR2* expression in cancer tissues was associated with improved overall survival in acute myeloid leukemia (AML) and Kidney Renal Clear Cell Carcinoma (KIRC) patients. Our results, showing that the upregulation of MHC class I by NK-derived IFN-γ leads to resistance to NK cytotoxicity, suggest that targeting IFN-γ responses might be a promising approach to enhance NK cell anti-cancer efficacy.

## Introduction

NK cells are crucial for cancer immune surveillance due to their robust effector function and intrinsic anti-cancer activity. The responses of NK cells to cancer or infected cells are determined by the integration of signals from activating and inhibitory receptors (1). Some cancer or stressed cells upregulate ligands for NK activating receptors, thereby stimulating NK cell responses. NK cells express various germline-encoded activating receptors, such as the Fc receptor CD16, natural-cytotoxicity-receptors (NKp30, NKp44, and NKp46) (2), receptors of the SLAM family (2B4 and SLAMF7), NKG2D, and co-stimulatory receptors such as CD2, DNAM-1, and CD28H (1, 3). Although NK cells express a broad array of receptors to recognize ligands on cancer cells, activation signaling alone is not sufficient to determine NK cell responses. NK cell activity is controlled by dominant inhibitory receptors for MHC class I, specifically, the receptor NKG2A–CD94 for HLA-E and inhibitory receptors of the killer-cell Ig-like receptor (KIR) family for HLA-C and some alleles of HLA-A and HLA-B. Signaling from inhibitory receptors locally blocks activation signaling and prevents NK cell activation from the beginning. Moreover, engagement of these inhibitory receptors by their respective MHC class I ligands protects NK cells from chronic stimulation and maintains them in a highly responsive state, a process termed “education” or “licensing” (4). NK cells can be activated by cancer cells that have upregulated the expression of ligands for NK cell activating receptors and down-regulated MHC class I expression.

Although NK cells can recognize a broad range of cancer cells *in vitro*, the contribution of their activity to immunotherapy for solid tumors has just begun to be appreciated (5, 6). Pre-clinical data have shown that NK cell depletion increases tumor growth (7). Accordingly, analysis of RNA-seq data from the TCGA database showed a strong correlation of NK cell infiltration with an improved patient survival rate in all cancer types (8, 9). Moreover, NK cells are critical for the beneficial responses observed in PD-1 and PD-L1 blockade in different murine cancer models (7). Clinical data also support a contribution of NK cells to favorable responses in checkpoint blockade therapy. An NK cell gene signature of metastatic melanoma patients showed a strong association between NK cell infiltration and increased overall survival, which could predict responsiveness to anti-PD-1 immunotherapy (10).

Exploiting the allogeneic activity of NK cells to treat leukemia has proved effective over decades of clinical work (11). Specifically, a haploidentical combination has been used to unleash the anti-leukemia activity of allogeneic NK cells during hematopoietic stem cell transplantation (HSCT) in leukemia patients. Briefly, the HSCT graft is usually selected from a family member who is identical for one HLA haplotype and mismatched for the other (11). Therefore, NK cell subsets for which HLA ligands for their KIRs are missing in the host will bypass inhibition and exert graft-versus-leukemia activity (11). T cells are extensively depleted from the graft to prevent graft-versus-host disease (GVHD). Incompatibility of inhibitory KIRs on graft NK cells with their MHC class I ligand from recipients is associated with a profound graft-versus-leukemia (GvL) effect and lower probability of relapse for AML patients (11–13).

Although the anti-leukemia activity of NK cells has long been recognized, the factors that dictate its efficacy have not been fully explored. To address this question, we carried out a CRISPR-cas9 genome-wide screen in the CML cell line K562 and identified genes that modulate its vulnerability to killing by NK cells. We determined that the NKp30 ligand B7H6 (14), which is encoded by *NCR3LG1*, is a dominant contributor to the NK-mediated killing of K562 cells. We also identified a potential negative regulation of anti-leukemia NK cell responses through MHC class I upregulation mediated by NK-derived IFN-γ, which might be targeted in cancer immunotherapy to enhance NK cell activity.

## Materials and Methods

### Reagents

Key reagents and their source are listed in Table 1.

**Table 1:** Key resources.

**Reagent** **type**	**Designation**	**Source**	**Identifier**	**Sub-class**	**Clone** **name**
Antibody	Anti-human CD107a FITC	BD Biosciences	555800	Mouse IgG_1_	H4A3
Antibody	Anti-human CD56 BV421	BD Biosciences	562751	Mouse IgG_2*b*_	NCAM16.2
Antibody	IFN-γ neutralization antibody	Mabtech	3420- 1N-500	Mouse IgG_2*a*_	MT111W
Antibody	Anti-human HLA-A, B, C APC	Biolegend	311409	Mouse IgG_2*a*_	W6/32
Antibody	Anti-human HLA-E BV421	Biolegend	342611	Mouse IgG_1_	sD12
Antibody	Anti-human B2M APC	Biolegend	316311	Mouse IgG_1_	2M2
CRISPR library	GeCKO V2 human CRISPR knockout	Addgene	1000000049		
Fluorescent Dye	PKH26 Red Fluorescent Cell Linker Kit	SIGMA	PKH26GL		
Fluorescent Dye	PKH67 Green Fluorescent Cell Linker Kit	SIGMA	PKH67GL		
Counting beads	123count eBeads™	ThermoFisher	01-1234-42		
Commercial kit	Dead Cell Removal Kit	Miltenyi Biotec	130-090-101		
Commercial kit	In-Fusion HD cloning kit	Clontech Laboratories	639648		
Commercial kit	EasySep Human NK Cell Enrichment Kit	STEMCELL Technologies	19055		
Commercial kit	EasySep™ Human CD56 Positive Selection Kit	STEMCELL Technologies	17855		
PCR polymerase	NEBNext® High-Fidelity 2X PCR Master Mix	New England Biolabs	M0541L		
Cytokine	Recombinant Human IFN-γ	PeproTech	300-02		

### Cells

Human NK cells were isolated from peripheral blood of healthy U.S. donors by negative selection (Stemcell Technologies). NK cells were resuspended in Iscove's modified Dulbecco's medium (IMDM; Gibco) supplemented with 10% human serum (Valley Biomedical) and used within 4 days. To obtain IL-2-activated NK cells, freshly isolated NK cells were co-cultured with irradiated autologous feeder cells in OpTimizer (Invitrogen) supplemented with 10% purified IL-2 (Hemagen), 100 units/mL recombinant IL-2 (Roche), and 5 μg/mL phytohemagglutinin (PHA, Sigma) and expanded in the same medium without PHA and feeder cells. The human erythroleukemia cell line K562 (American Type Culture Collection, Manassas, VA) were cultured in RPMI 1640 supplemented with 2 mM L-glutamine and 10% fetal bovine serum (FBS; Atlanta Biologicals).

### Plasmids and Lentivirus Production

gRNAs targeting individual genes were synthesized from IDT (Integrated DNA Technologies), annealed as previously described (15) and cloned into the BsmBI restriction sites of the LentiGuide-Puro vector (Addgene, 52963). Cloning was performed using the In-Fusion HD cloning kit (Clontech). The lentivirus production procedure has been described previously (3, 16).

### Genome-Wide Cancer Vulnerability and Resistance Screen

K562 cells were transduced with LentiBlast-Cas9 and selected by 10 μg/ml blasticidin to obtain stable expression. GeCKO V2 human CRISPR knockout library (Addgene) was transduced into Endura™ Electrocompetent Cells (Lucigen, 60242-1) by electroporation using a Bio-Rad Gene Pulser (Bio-Rad) as described (16). Expanded CRISPR plasmid libraries were purified by Maxi-Prep (Qiagen) and used for lentivirus production (3, 16). Lentivirus titer was determined as previously described (16). Cas9-expressing K562 cells were transduced with GeCKO V2 lentivirus libraries at a low MOI of 0.3 and selected in puromycin for 7 days. 50 × 10^6^ transduced K562 cells were incubated with IL-2-activated NK cells at an E to T ratio of 0.3:1. Percentages of surviving K562 cells were monitored. If needed, extra NK cells were added until only 10% of K562 cells had survived. To recover surviving K562 cells, dead cells were removed by Dead Cell Removal Kit (Miltenyi Biotec) followed by depletion of NK cells using EasySep™ Human CD56 Positive Selection Kit (Stemcell Technologies). In screens with low selection pressure, recovered K562 cells were refreshed in complete media for 48 h before genomic DNA extraction. To achieve higher selection pressure, recovered K562 cells were further cultured up to 50 × 10^6^ cells, which were selected again by two rounds of co-incubation with NK cells. Control K562 cells were kept in the same culture conditions without exposure to NK cells. Two biological repeats were performed in the screen under low selection pressure, and two technical repeats were performed in the screen with high selection pressure. Genomic DNA extraction and gRNA cassette amplification were carried out as described previously (16). Amplified libraries were multiplexed and analyzed on a NextSeq 500 (Illumina) with 75-bp single-end reads. Analysis of gRNA enrichment/ depletion was performed using MAGeCK-VISPR V 0.5.4 (17). Briefly, this pipeline calculates the individual sgRNA read counts in libraries from both control and surviving K562 cells. After normalizing to the total reads of each library, the read counts of individual gRNAs are compared between control and surviving K562 cells. Compared to control K562 cells, read counts of enriched gRNAs increase in surviving K562 cells, whereas read counts of depleted sgRNAs decrease in surviving K562 cells. The score of each gene represents the normalized fold changes of all gRNAs targeting this gene. Pathway analysis was carried out using Ingenuity Pathway Analysis (IPA, QIAGEN Bioinformatics). Protein network analysis was performed using STRING (v11.0).

### Flow Cytometry

For immune staining before flow cytometry, cells were incubated with premixed fluorophore-conjugated antibodies at 4°C for 30 min. Cells were washed after staining and analyzed on an LSR II (BD Biosciences) or LSRFortessa X-20 (BD Biosciences). Data were analyzed with FlowJo (FlowJo, LLC).

### Cytotoxicity Assay

Cytotoxicity assays were performed using a flow-based method. Briefly, K562 cells were labeled with PKH67-green or PKH26-red membrane dye and washed with complete media. IL-2 activated NK cells were co-incubated with pre-labeled K562 cells for the indicated time at 37°C in IMDM media supplemented with 10% FBS. The number of surviving K562 cells was calculated using counting beads by flow cytometry. Occasionally, a 2nd round of NK cells was added, as specified for each assay.

### Degranulation Assay

Degranulation assays were performed using resting human NK cells. Briefly, NK cells were co-incubated with target cells for 2 h at 37°C and stained with Live/Dead-NIR (Thermo Fisher), anti–CD56-Bv421 (BD 562751) and anti–CD107a-FITC (BD 555800). Samples were analyzed by flow cytometry.

### TCGA-LAML Survival Curve Analysis

Kaplan-Meier curves were generated using GraphPad PRISM V8. Patient data from the TCGA acute myeloid leukemia (LAML) database were downloaded from https://xena.ucsc.edu/. Patient samples were separated using the 33rd and 66th percentile values of *NKG7* expression to threshold, and the partitioning of patient samples with *IFNGR2* used the 40th and 60th percentile values to threshold. Similarly, TCGA Kidney Renal Clear Cell Carcinoma (KIRC) patient data were downloaded from https://xena.ucsc.edu/ and partitioned into samples with *IFNGR2*, using the 33th and 66th percentile values to threshold. The log-rank (Mantel-Cox) test was used to calculate a *P*-value for overall patient survival.

### Statistical Analysis

Statistical analysis was performed with GraphPad PRISM V8. Data were represented as mean with SEM and compared by paired ANOVA test.

### Human Donors

Peripheral blood samples from healthy U.S. adults were obtained from the NIH Department of Transfusion Medicine in accordance with the Belmont Report, under an NIH Institutional Review Board-approved protocol (99-CC-0168) with informed written consent.

## Results

### Loss of *NCR3LG1* Expression Protects K562 Cells From NK-Dependent Lysis

**Figure 1:**
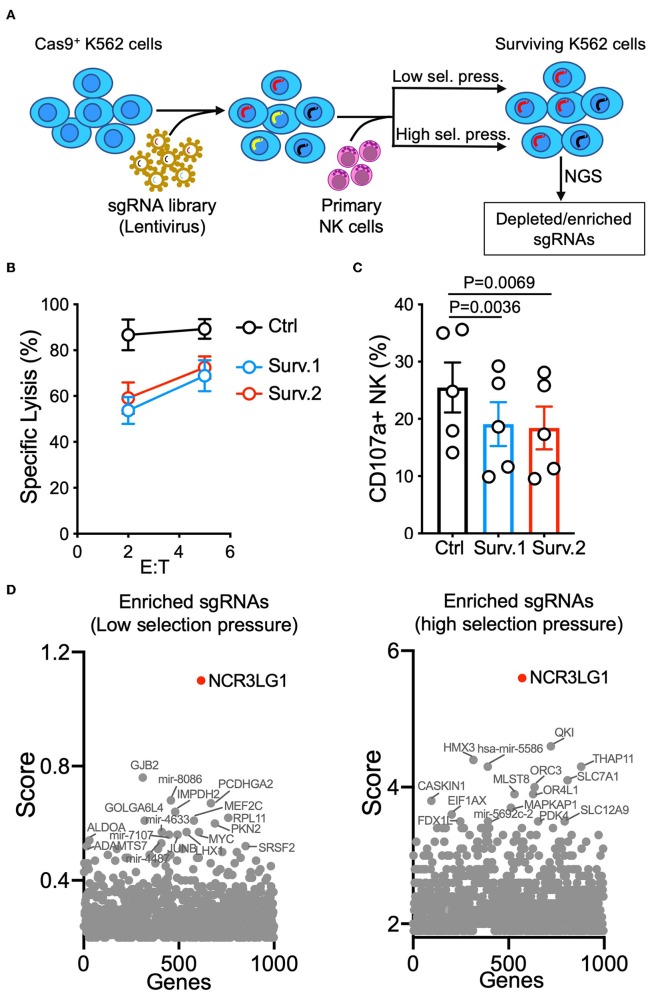
Genome-wide vulnerability CRISPR screen in K562 cells. **(A)** Diagram of experimental design. K562 cells expressing Cas9 were transduced with a sgRNA library and co-incubated with IL-2-activated human NK cells. For low selection pressure, K562 cells were co-incubated with NK cells until only 10% of them survived. For high selection pressure, three rounds of co-incubation with NK cells were performed. Distributions of sgRNAs in K562 cells that survived were analyzed by next-generation sequencing. **(B)** NK cytotoxicity assay with control or surviving K562 cells obtained in two parallel cultures (Surv.1 and Surv.2). K562 cells recovered after high selection pressure were refreshed for at least 48 h in fresh complete media before cytotoxicity assays. Control cells were K562 cells (transduced with Cas9 and a CRISPR library) cultured in parallel without exposure to NK cells (*n* = 4). **(C)** Degranulation of resting NK cells from individual donors in response to control or K562 cells that survived (paired ANOVA, *n* = 5). **(D)** Top enriched sgRNAs in cancer vulnerability screen with low selection pressure (left) and high selection pressure (right). Normalized log fold changes (score) of genes were calculated using MAGeCK-VISPR.

K562 is one of the most commonly used target cell lines for NK cell functional assays due to their high sensitivity to NK-mediated lysis. K562 cells were transfected and selected for stable expression of Cas9 before transduction with the GeCKO V2 CRISPR-knockout lentivirus library at a low multiplicity-of-infection (MOI of 0.3) (16). Selection by NK-mediated cytotoxicity was performed by either a single round of co-incubation with primary IL-2-activated NK cells (lower selection pressure) or three rounds of co-incubation for higher selection pressure (Figure 1A). The resistance of surviving K562 cells was confirmed by a lysis assay (Figure 1B). Compared to control K562 cells, surviving K562 cells were less sensitive to NK-mediated lysis and induced degranulation by fewer NK cells (Figures 1B,C), suggesting that co-incubation with NK cells enriched K562 cells that had been edited to escape NK-mediated killing. Analysis of gRNAs enriched by both low and high selection pressure revealed that loss of expression of *NCR3LG1* (B7H6), a ligand for natural cytotoxicity receptor *NCR3* (NKp30), protected K562 cells from lysis (Figure 1D). Although K562 cells express ligands for several other NK cell activating receptors (18), our functional screen showed that B7H6 is a major contributor to the killing of K562 cells by NK cells.

### Genes in the IFNGR-JAK-STAT and Antigen Presentation Pathways Protect K562 Cells From Lysis by NK Cells

**Figure 2:**
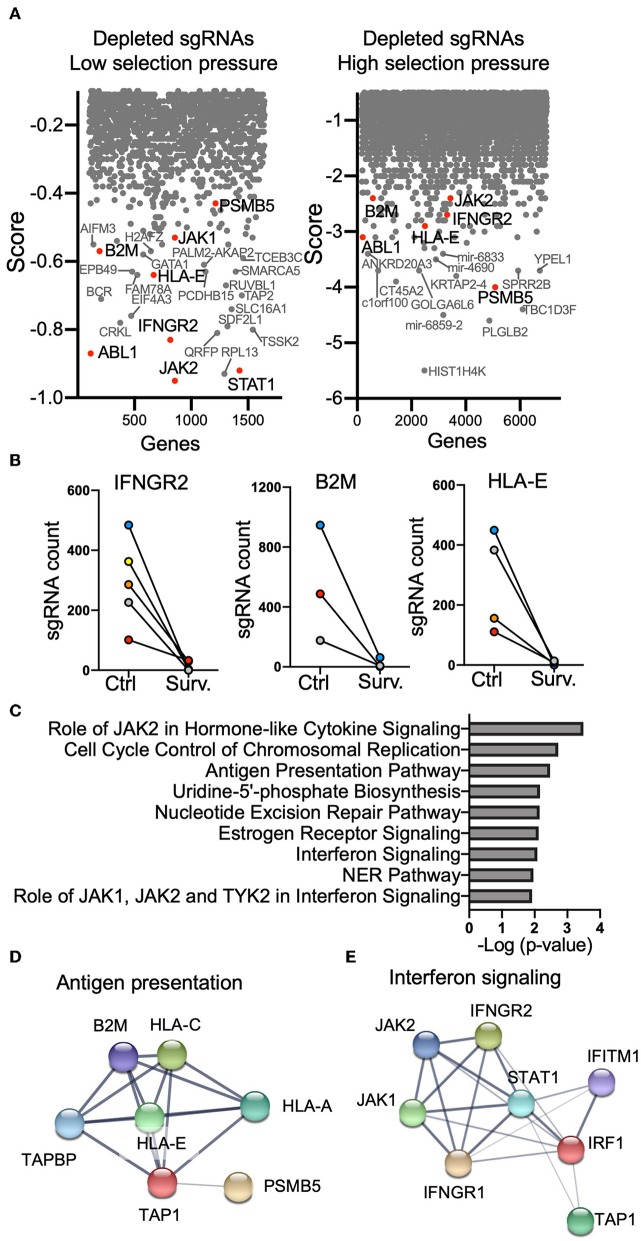
Genome-wide resistance CRISPR screen in K562 cells. **(A)** Top depleted sgRNAs in cancer vulnerability screen. Normalized log fold changes (score) of genes were calculated using MAGeCK-VISPR. Selected genes identified in both low and high selection pressure are in red. **(B)** sgRNA counts for a few selected genes. **(C)** Pathway analysis of top depleted sgRNAs. **(D,E)** Protein network analysis of top depleted sgRNAs in the antigen presentation pathway **(D)** and interferon signaling pathway **(E)**.

Analysis using the MAGeCK-VISPR pipeline (17) revealed that depleted gRNAs were predominantly targeting genes in the antigen-presentation pathway and the IFNGR-JAK-STAT pathway in screens with both higher and lower selection pressure, indicating that knocking out genes in these two pathways increased the vulnerability of K562 cells to NK-mediated lysis (Figure 2A). Multiple gRNAs targeting each gene in the IFNGR-JAK-STAT pathway were depleted in surviving K562 cells (Figure 2B). Moreover, loss of expression of the oncogenic tyrosine kinase *ABL1* also sensitized K562 cells for killing (Figure 2A). K562 cells are positive for the Philadelphia chromosome (Ph)-specific fusion gene *BCR-ABL* (19, 20), which is found in almost all CML patients. Susceptibility of K562 cells to apoptosis can be increased by downregulation of *BCL-ABL* expression (20). Pathway analysis and protein network analysis further confirmed that the expression of genes related to antigen-presentation and interferon signaling conferred protection to K562 cells from killing by NK cells (Figures 2C–E). NK cells are one of the major sources of IFN-γ in the immune system. NK cell activation is dominantly controlled by inhibitory receptors for MHC class I, the expression of which can be upregulated by IFN-γ (1). Therefore, a potential negative feedback loop of NK cell activity is that IFN-γ secreted by NK cells upon stimulation by cancer cells upregulates MHC class I on cancer targets and subsequently attenuates NK-mediated lysis.

### IFN-γ Produced by NK Cells Induces MHC Class I Expression and Protection of K562 Cells

**Figure 3:**
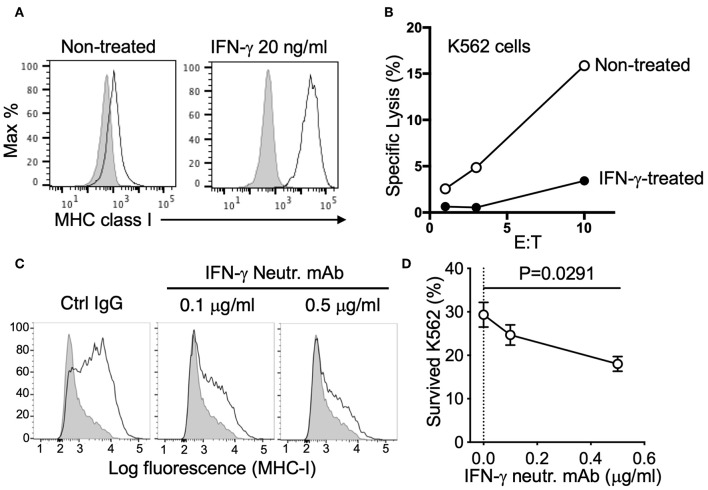
Resistance of K562 cells conferred by NK-derived IFN-γ. **(A)** Expression of MHC class I (mAb W6/32) on K562 cells, either non-treated or treated with 20 ng/ml IFN-γ for 16 h. **(B)** NK-mediated lysis of control or IFN-γ-treated K562 cells (representative of two experiments). **(C)** MHC class I expression on K562 cells after co-incubation with NK cells (E:T = 0.3:1) for 16 h in the presence of control IgG (mIgG2a) or IFN-γ neutralizing antibody (MT111W). **(D)** K562 cells that survived after two consecutive incubations with NK cells in the presence of control IgG (mIgG2a) or IFN-γ neutralizing antibody (MT111W). Pre-labeled K562 cells were exposed to NK cells at an E:T of 0.3:1 for 16 h in the presence of the indicated IgGs. The same number of NK cells was added for the second round of selection and incubated for 24 h. K562 cells that survived were quantified by flow cytometry (paired ANOVA, *n* = 3).

To clarify this potential negative regulation, we tested the response of K562 cells to both recombinant and NK-derived IFN-γ. K562 are sensitive target cells for NK cells due in part to their low/negative expression of MHC class I. However, expression of MHC class I was dramatically upregulated by treatment with IFN-γ (Figure 3A). Killing assays revealed that IFN-γ treatment conferred protection to K562 cells by reducing NK-mediated specific lysis (Figure 3B). Similarly, MHC class I expression was strongly increased after co-incubation with NK cells, even at the low effector to target (E to T) ratio of 0.3:1 (Figure 3C). To block MHC class I upregulation by NK-derived IFN-γ, we utilized an IFN-γ neutralizing antibody, MT111W, which had been proved to be effective for neutralization in a previous study (21). The response to IFN-γ of K562 cells and MHC class I upregulation was efficiently blocked by adding MT111W during NK–K562 co-incubation (Figure 3C). Notably, IFN-γ neutralization efficiently sensitized K562 cells for killing by NK cells (Figure 3D).

### Interference With the IFNGR-JAK-STAT and Antigen Presentation Pathways Sensitizes Cancer Cells for NK-Mediated Killing

**Figure 4:**
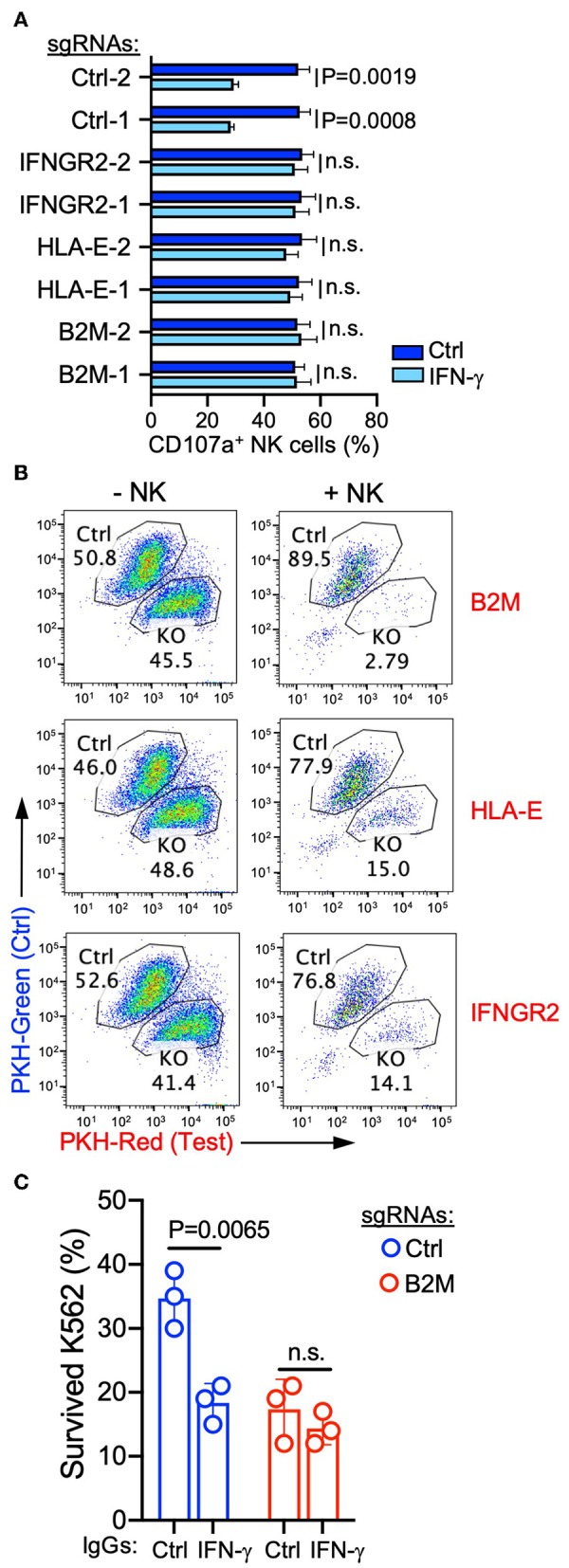
Interference with the IFNR-JAK-STAT and antigen presentation pathways sensitized K562 cells for NK-mediated killing. **(A)** Degranulation of resting NK cells induced by K562 cells expressing the indicated sgRNAs. K562 cells were either non-treated or pre-treated with 20 ng/ml IFN-γ for 16 h before incubation with NK cells for 2 h (*n* = 4; unpaired *t*-test; n.s., non-significant). **(B)** Killing assay to compare the sensitivity of K562 cells expressing control sgRNA (pre-labeled with PKH-green) and sgRNAs targeting the indicated genes (pre-labeled with PKH-red). K562 cells were co-incubated with NK cells for 48 h at an E to T ratio of 0.3:1, followed by the addition of the same number of NK cells and incubation for 24 h. K562 cells that survived were quantified by flow cytometry (representative of two experiments). **(C)** Lysis of K562 cells expressing control or B2M sgRNA in the presence of 1 μg/ml control IgG (mouse IgG2a) or IFN-γ neutralizing antibody (MT111W). K562 cells were co-incubated with NK cells at an E to T ratio of 0.3:1 for 16 h, followed by the addition of the same number of NK cells and incubation for 24 h (*n* = 3; unpaired *t*-test; n.s., non-significant). Control IgG and IFN-γ neutralizing antibody were present during both rounds of incubation.

To further confirm that targeting the antigen-presentation pathway and IFNGR signaling pathway can increase the susceptibility of cancer cells to NK-mediated lysis, we transduced individual gRNAs targeting each selected gene from these two pathways (Figure S1A). Induction of B2M expression by recombinant IFN-γ was decreased by expression of *B2M* or *IFNGR2* gRNAs in K562 cells, and IFN-γ-induced HLA-E expression was abolished by both gRNAs targeting *HLA-E* (Figure S1B), indicating the high gene-editing efficacy of these selected gRNAs. Similarly, the expression of *B2M*-targeting gRNAs also abrogated responses to NK-derived IFN-γ, and B2M expression remained negative after co-incubation with NK cells (Figure S1C). Compared to non-treated cells, IFN-γ treated control K562 cells induced degranulation by fewer NK cells (Figure 4A). However, K562 cells expressing gRNAs targeting *B2M, HLA-E*, or *IFNGR2* lost this response to IFN-γ (Figure 4A) and induced comparable NK cell degranulation after treatment with IFN-γ (Figure 4A). Similarly, in cytotoxicity assays with a mixture of control and knockout K562 cells pre-labeled with different colors and co-incubated with NK cells, the expression of gRNAs targeting *B2M, HLA-E*, or *IFNGR2* increased the susceptibility of K562 cells to killing (Figure 4B). Moreover, IFN-γ neutralization increased the sensitivity of control K562 cells for NK-mediated lysis, whereas the killing of K562 cells expressing *B2M* gRNA remained the same in the presence of IFN-γ neutralizing antibody (Figure 4C). These results confirmed that NK-derived IFN-γ negatively regulated NK-mediated killing through the up-regulation of MHC class I and B2M on target cells.

### Low Expression of *IFNGR2* in Cancer Tissues Correlates With Better Survival of AML and KIRC Patients

**Figure 5:**
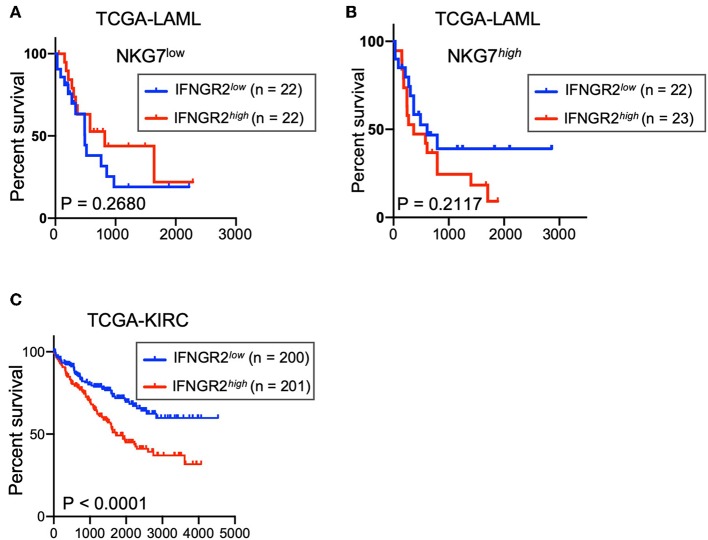
Low abundance of *IFNGR2* mRNA in cancer tissues correlated with better survival of AML and KIRC patients. **(A,B)** Survival of patients from the TCGA acute myeloid leukemia (LAML) database partitioned by *IFNGR2* and split by NKG7 expression. **(C)** Survival of patients from the TCGA kidney renal clear cell carcinoma (KIRC) database, partitioned by *IFNGR2*.

We explored the potential contribution of this negative feedback to the survival rate of leukemia patients from the TCGA acute myeloid leukemia (LAML) database. Despite the relatively small size of the patient cohort, a trend of better overall survival was observed in AML patients with cancer tissues that had lower expression of *IFNGR2* and higher expression of *NKG7* (Figures 5A,B). *NKG7* is an NK signature gene that is highly correlated with NK scores (9). Furthermore, a stronger association of low *IFNGR2* mRNA abundance in tumors with improved overall survival was observed in patients in the TCGA Kidney Renal Clear Cell Carcinoma (KIRC) database (Figure 5C). The result of our analysis implied that higher expression of *IFNGR2* in cancer cells and its downstream signaling is unfavorable in AML and KIRC patients, potentially due to constraints on NK cell activity. Targeting IFN-γ signaling by IFN-γ neutralizing antibodies or other approaches might promote NK cell anti-cancer responses and improve the survival rate of cancer patients.

## Discussion

IFN-γ regulates the immunogenicity of cancer cells by upregulating MHC class I and promoting antigen presentation in cancer cells. Therefore, IFN-γ secretion is usually recognized as favorable in cancer patients due to improved susceptibility of cancer cells to cytotoxic T cells. In accordance with this, a different genome-wide CRISPR screen found that genes in the IFN-γ signaling pathway and antigen presentation pathway were crucial for lysis of cancer cells by cytotoxic T cells (22). Loss of expression of these genes was dominantly associated with tumor immune evasion (22). Similarly, in CRISPR screens with two different murine cancer cell lines, MC38 colon adenocarcinoma and B16 melanoma cells, which had been engineered to present chicken ovalbumin (Ova) antigen, IFN-γ signaling and antigen presentation were also found to be critical for cytotoxic T cell-mediated killing (23). However, increased MHC class I expression on cancer cells by IFN-γ can engage inhibitory receptors on NK cells and restrain NK-mediated anti-cancer activity. The dark side of IFN-γ, which promotes tumor immune evasion and growth, has been noticed in experimental and clinical studies (24). IFN-γ signaling and upregulation of non-classical MHC class I have been implicated in metastasis, immune evasion, and resistance to NK cells in a variety of cancers (24). Moreover, a similar immune escape associated with increased expression of MHC class I was observed in haploidentical NK-cell therapy in primary chemotherapy-refractory or relapsed high-risk myelodysplastic syndrome (MDS), secondary AML (MDS/AML), and *de novo* AML patients (25). These patients received adoptive transfer of IL-2–activated haploidentical NK cells, and an objective response rate of 38% was achieved (25). However, upregulation of classical MHC class I and HLA-E was observed on the residual and relapsed blast cells in responding patients. Retreatment under the same protocol was shown to be ineffective in relapsed patients, indicating immune escape mediated by upregulation of MHC class I, potentially induced by donor NK-derived IFN-γ (25).

As a multi-functional cytokine, IFN-γ induces the expression of other molecules besides MHC class I, such as ICAM-1 and PD-L1. In another CRISPR screen in K562 cells, using the NK cell line NK-92 as an effector for killing, the IFN-γ pathway was found to contribute, through upregulation of ICAM-1, to lysis of K562 cells by NK-92 cells (26). The discrepancy of this screen with our results is unclear. It is possible that the hierarchy of other factors that contribute to tumor lysis mediated by NK-92 cells and by primary NK cells is different and that the absence of inhibitory KIR on NK-92 cells is a contributing factor. Induction of PD-L1 by IFN-γ can also inhibit NK and T cell activity through checkpoint receptor PD-1 and protect tumor cells from killing (7). Therefore, other factors, such as upregulation of PD-L1 on tumor cells, could contribute to the negative correlation between IFNGR2 expression and the overall survival of AML and KIRC patients.

We have shown that interference with the IFN-γ signaling pathway and antigen presentation pathway sensitized leukemia cells for NK-mediated lysis. A recent genome-wide CRISPR screen in mouse B16-F10 melanoma cells revealed a similar role of the IFNGR-JAK-STAT pathway and upregulation of MHC class I in protecting tumor cells from killing by mouse NK cells (27), indicating that this negative feedback might be a universal mechanism for tumor immune evasion. Therefore, blockade of IFN-γ signaling in cancer cells by either neutralizing IFN-γ or blocking IFN-γ receptors might improve immunotherapy using NK cell-dependent anti-cancer responses. This strategy could be combined with adoptive transfer of haploidentical NK cells or chimeric-antigen-receptor (CAR)-expressing NK cells.

## Data Availability Statement

CRISPR screen datasets are available under the accession number GEO: GSE139313 (https://www.ncbi.nlm.nih.gov/geo/query/acc.cgi?acc=GSE139313). All other raw data supporting the conclusions of this manuscript will be made available by the authors, without undue reservation, to any qualified researcher.

## Ethics Statement

The studies involving human participants were reviewed and approved by NIH Institutional Review Board-approved protocol (99-CC-0168). The patients/participants provided their written informed consent to participate in this study.

## Author Contributions

XZ conceived the study, carried out the experiments, analyzed the data, and wrote the paper. DV performed data analysis. EL conceived and supervised the study and wrote the paper.

### Conflict of Interest

The authors declare that the research was conducted in the absence of any commercial or financial relationships that could be construed as a potential conflict of interest.
