# Supplementary material for: Genome-Wide CRISPR Screen Reveals Cancer Cell Resistance to NK Cells Induced by NK-Derived IFN-γ
Source: Front Immunol. 2019 Dec 11;10:2879. doi: 10.3389/fimmu.2019.02879 (PMC6917608; doi:10.3389/fimmu.2019.02879)
Supplement: Supplementary file 1 [file Data_Sheet_1.pdf]

Supplementary Material

Supplementary Figure

A

sgRNA	Numbering in GeCKO V2 library	Sequence
B2M-1	B_03944	CGTGAGTAAACCTGAATCTT
B2M-2	A_03946	CAGTAAGTCAACTTCAATGT
HLA-E-1	B_22615	CCGAGCCCGTCACCCTGAGA
HLA-E-2	A_22642	ACCGGGAGACACGGAGCGCC
IFNGR2-1	A_31002	ACGGCCGACATCATGTCCAT
IFNGR2-2	B_23681	TGACATCGCTGATACCTCCA
Ctrl-1	A_14986	TCCTGCCAAGAAACACCCTT
Ctrl-2	A_15033	ATCAAAGTGTCTGACTTATT

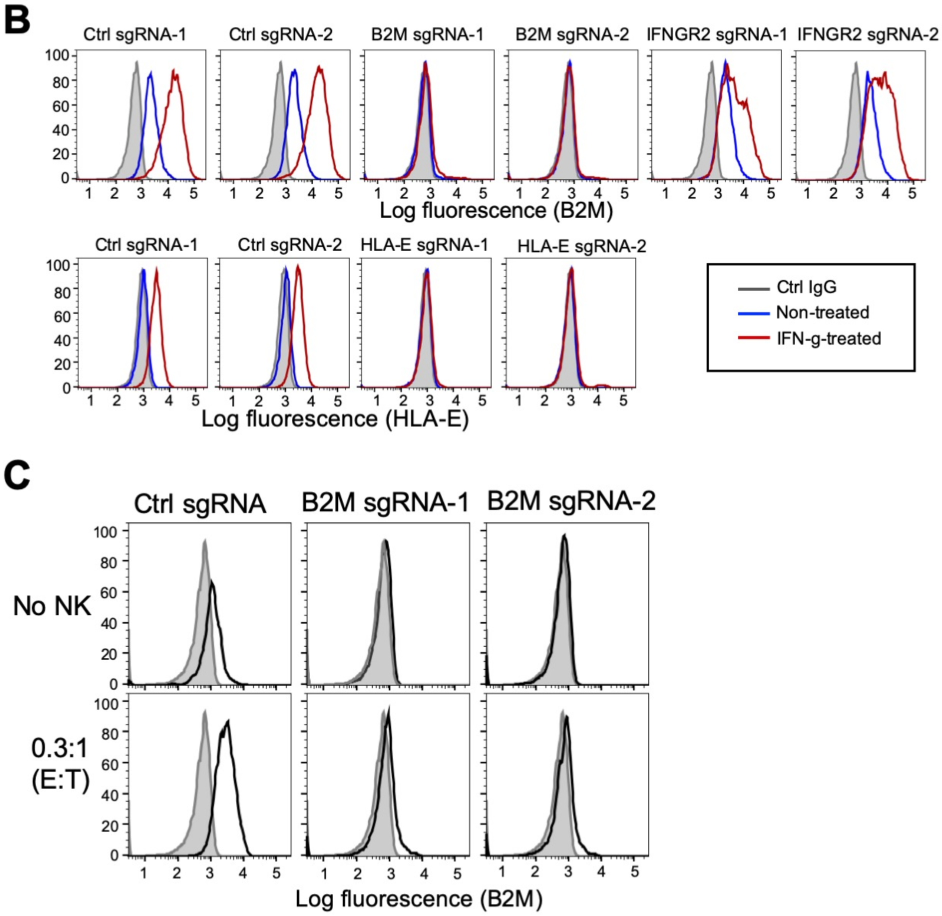

Figure S1. Related to Figure 4. (A) Sequence of selected gRNAs. (B) Histograms showing the expression of B2M or HLA-E on K562 cells expressing the indicated sgRNAs. Cells were either non-treated (blue lines) or treaded with 20 ng/ml IFN-γ for 16 hours (red lines) before the staining. (C) Flow staining for control IgG (shaded) or B2M antibody (black lines) on K562 cells that have been transduced with control sgRNA or B2M sgRNA. K562 cells were non-treated or co-incubated with NK cells (E: T = 0.3:1) for 16 hours before staining.
